# Real-World Testing of an Artificial Intelligence Conversational Agent as an Early Intervention and Support Tool in the Mental Health Referral Care Pathway: A Mixed-Methods Study

**DOI:** 10.1177/00207640251415507

**Published:** 2026-03-05

**Authors:** Edward Meinert, Madison Milne-Ives, Emma Taylor, Becky Inkster, Alina Paik, Ananya Ananthakrishnan, Martin Orr, Ceire Costelloe, Rohit Shankar

**Affiliations:** 1 Translational and Clinical Research Institute, Newcastle University, Newcastle upon Tyne, UK; 2 Gnosis Health Limited, Newcastle Helix, Newcastle Upon Tyne, UK; 3 Department of Primary Care and Public Health, School of Public Health, Imperial College London, UK; 4 Wysa, London, UK; 5 Department of Psychiatry, University of Cambridge, UK; 6 Department of Psychology, Central North West London NHS Foundation, UK; 7 Division of Clinical Studies, Institute of Cancer Research, London, UK; 8 Peninsula Medical School, Faculty of Health, University of Plymouth, UK; 9 Cornwall Intellectual Disability Equitable Research unit, Cornwall Partnership NHS Foundation Trust, Bodmin, UK

**Keywords:** artificial intelligence, mental health, telemedicine, randomized controlled trial

## Abstract

**Background:**

The incidence of mental health concerns is growing, and demand for support is exceeding service capacity. Digital tools can provide additional support but risk causing harm if not delivered safely.

**Aims:**

We aimed to establish real-world evidence of the impact of an artificial intelligence-based mental health conversational agent (Wysa) on depression and anxiety in patients waiting for Talking Therapies treatment.

**Methods:**

A mixed-methods randomized controlled trial was conducted with patients referred to Talking Therapies in the Central and Northwest London NHS Foundation Trust. The primary outcome was change in depression severity over 12 weeks between groups; secondary outcomes included anxiety severity, quality of life, safety, engagement, and app usage. Comparative analyses used linear regression; thematic analysis was conducted on qualitative data.

**Results:**

2,161 patients were screened, 625 were invited, 99 consented, and 76 were randomized (2:1). Thirty patients were lost to follow-up. Descriptive analysis found that mean differences in depression were similar between arms, but with large standard deviations (*M* = 2.62, *SD* = 5.07 and 6.56 for Wysa; *M* = 2.59, *SD* = 4.38 and 3.82 for control). Results were similar for secondary outcomes. Wysa was potentially helpful, easy to use, and appreciated as an accessible source of support, but limitations with the conversational agent negatively affected engagement.

**Conclusions:**

Although sample size limited the analysis, participant feedback highlighted its potential to supplement clinical services. Our study findings suggest that the change of depression score is similar in both arms thus indicating that there is no evidence that Wysa treats depression in this study. However, limited sample size could have influenced this. Key lessons to improve the quality of effectiveness studies of digital health technologies were identified.

## Introduction

Mental health concerns can have significant negative impacts (Mental Health, n.d.; World Health Assembly, 2012), but many people do not receive sufficient support (Wainberg et al., 2017). Approximately 75% of people who could benefit from support do not receive it, resulting in approximate costs of £105.2 billion/year (Alonso et al., 2018; The Five Year Forward View for Mental Health, 2016). There are also inequalities in access (Bansal et al., 2022; Lowther-Payne et al., 2023) and often long wait times (Community Mental Health Survey 2020, n.d., 2020). Digital technologies could provide more accessible support (Tal & Torous, 2017) and mitigate negative outcomes and unhealthy coping strategies (The Improving Access to Psychological Therapies Manual, 2019). Implementing early automated support could reduce face-to-face assessments and demand on emergency services by providing an alternative treatment pathway. Good quality evidence that such technologies are safe and effective (Abd-Alrazaq et al., 2020; Lecomte et al., 2020; Skorburg & Yam, 2022) is limited. There is some evidence that mental health apps can influence health outcomes (Arean et al., 2016; Torous et al., 2020), but recent reviews have found limited evidence of impact (Marshall et al., 2020; Neary & Schueller, 2018; Torous et al., 2018; Wang et al., 2018), particularly for AI-enabled apps (Milne-Ives et al., 2022). AI interventions also have ethical concerns due to potential harms (Sun et al., 2023; Torous et al., 2018). Conversational agents (CAs) are computer programs designed to simulate human conversation using natural language and may provide inappropriate or dangerous advice or increase emotional dependence on the intervention at the expense of human connection (Hamdoun et al., 2023; Laestadius et al., 2022). AI apps may not sufficiently protect privacy or use algorithms that are generalizable to different patient groups (Burr et al., 2020; Hamdoun et al., 2023; Ray et al., 2022; Torous et al., 2018), and they risk exacerbating the “digital divide,” the gap between those who understand and make use of digital technologies and those who do not, contributing to a divide between those leveraging technology for advancement and those being left behind (Skorburg & Yam, 2022). This emphasizes the necessity of rigorously evaluating their effectiveness and safety, to ensure that there is limited opportunity for these technologies to cause harm.

This study aimed to examine the efficacy of real-world use of an AI-enabled self-help CA app (Wysa) as a supplementary tool for patients waiting for mental health support through the Talking Therapies program (formerly Improving Access to Psychological Therapies (IAPT)). The primary hypothesis was that access to Wysa would improve symptoms of depression compared to a waitlist control; secondary hypotheses also predicted improvement for symptoms of anxiety and health-related quality of life. To provide a more in-depth understanding of real-world use, the study also aimed to explore engagement with and perceptions of Wysa.

## Methods

### Study Design

This single-center, parallel-group, randomized control trial (RCT) used mixed methods to compare the intervention group against a waitlist control group (Supplemental Appendix 1) (Taylor-Powell & Henert, 2008). Participants were allocated 2:1 (Wysa: control) and followed up over 12 weeks while receiving standard care. The funding commencement date was initially agreed as 28 June 2021. Following the CONSORT-AI checklist (Supplemental Appendix 2) (X. Liu et al., 2020), the study was approved on 25 May 2022 by the London – Stanmore Research Ethics Committee (22/PR/0467) but there were delays in HRA approval (14 October 2022) and site set-up. Recruitment took place between 13 December 2022 and 27 April 2023, lasting 4.5 months rather than the planned 7 months, as the funder end date was 30 August 2023. The protocol was registered on ClinicalTrials.gov (NCT05533190) on 5 September 2022 and ISRCTN (14644939) on 7 December 2022.

### Participants and Recruitment

Participants referred to three Talking Therapy services (participants on a waiting list for psychosocial intervention) within the Central North West London (CNWL) NHS Foundation Trust were screened for eligibility. The psycho-social interventions on offer at the NHS Trust include Cognitive Behavioral Therapy, Guided Self-Help, Counseling, Interpersonal Therapy, Dynamic Interpersonal Therapy, and psycho-educational groups and workshops. The specific therapy offered depends on individual recovery goals and the nature of their difficulties.

Table 1 outlines eligibility criteria.

**Table 1.** Inclusion and Exclusion Criteria.

Inclusion criteria	Exclusion criteria
18 years old or older	Previous and current major mental illness
Proficient in English	Current psychosis or a history of psychotic symptoms within 6 months
Willing and able to provide informed consent	Suicidal ideation
Referred or self-referred to IAPT with mild to moderate health concerns	Significant cognitive disorders, noted neurodevelopmental conditions, or personality disorder diagnosis
Owned a device capable of supporting Wysa	Patient Health Questionnaire (PHQ-9) or General Anxiety Disorder tool (GAD-7) score >15 (indicating moderately severe or severe symptoms)
	Under the care of specialized mental health services in the last 2 years
	Previously failed Talking Therapies
	Referrals for a specialized assessment beyond the standard clinical pathway
	Incapable of self-consent
	In a dependent/unequal relationship with any team member

### Randomization and Masking

A masked computer randomization algorithm (Sealed Envelope [Sealed Envelope, n.d.]) ensured the groups were balanced on gender, age, and iaptus triage PHQ-9 score. Randomization was encrypted to blind the researchers; the qualitative team was un-blinded for interviews.

### Intervention

Intervention group participants received an SMS link to download the full-access version of Wysa [version 0.1.4.0], a self-help tool for mental health that has been rated highly on privacy and security (*Privacy Not Included: A Buyer’s Guide for Connected Products, n.d.; Wysa, n.d.). The app asks users to provide information to personalize recommendations and collects routine outcome data aligned to existing service measures (The Improving Access to Psychological Therapies Manual, 2019) (PHQ-9 [Kroenke et al., 2001], GAD-7 [Spitzer et al., 2006]). Support is provided using AI-enabled tools – with natural language processing, conversation engines, a predetermined content library, and evidence-based therapeutic exercises and videos. Data is pushed directly into the patients’ electronic patient record (iaptus) and can generate automated safety alerts.

### Outcomes

The primary outcome was change in depression severity from baseline to 12 weeks between groups (PHQ-9 [Kroenke et al., 2001]). Secondary outcomes – anxiety severity (GAD-7 [Spitzer et al., 2006]) and health related quality of life (EQ-5D-5L [Herdman et al., 2011]) – were assessed similarly. App data captured a tally of exercises completed, sessions completed, and messages to Wysa. Interviews were structured based on the Theoretical Framework of Acceptability (TFA) (Sekhon et al., 2017) and a multifaceted conceptualization of engagement (Cole-Lewis et al., 2019; Kelders et al., 2020; O’Brien, 2016; Perski et al., 2017; Yardley et al., 2016) (Supplemental Appendix 3).

### Data Collection Procedure

An electronic data capture system (Castor Research Inc.) was used to collect and store data. Pre-randomization, patients completed a baseline questionnaire. Post-randomization, patients were called to identify technical issues or adverse events (3 weeks) and re-sent the questionnaire (12 weeks). Participants were randomly selected for semi-structured interviews in June 2023 and invited as they completed their intervention periods. Interviews were conducted via Microsoft Teams between 22 June and 25 July 2023. App use data was downloaded from Wysa servers. Demographic data and PHQ-9 and GAD-7 scores were collected from iaptus.

### Sample Size

A power calculation based on previous research (Inkster et al., 2018) indicated that a clinically meaningful effect size (a difference of at least 2 points on the PHQ-9 score) could be detected with 80% power with a sample size of 393. This sample size was increased to 480 to allow for 20% attrition.

Twenty participants were selected using stratified random sampling (by gender, ethnicity, and age) for semi-structured interviews. Deprivation levels (above or below median) and starting PHQ-9 levels (mild or moderate) were reviewed to ensure sufficient diversity; this did not require any adjustments. A backup list was generated by matching primary participants with demographically-similar remaining participants.

### Data Analysis

Descriptive statistics assessed baseline comparability and the distributions of the primary and secondary outcomes. Linear regression was used for comparative analysis of PHQ-9 and GAD-7. A within-item average for PHQ-9 and GAD-7 was taken when scores from questionnaires and iaptus differed. Because of the small sample size, the study statistician determined that the planned imputation of missing data was not advisable; missing values were excluded in descriptive analyses. *Post-hoc* regression was carried out for individuals with PHQ-2 scores (the sum of the first questions from the PHQ-9) ⩽5 and ⩽4 at baseline. Health-related quality of life was obtained via the “eq5d” package in R: this package transforms ordinal survey responses into a single index. Number of treatment sessions during the trial was captured as a post-treatment variable; controlling for this variable effectively regresses the component of the treatment effect that does not vary with the number of sessions onto the outcome measures (Lovell et al., 2008).

Qualitative data was analyzed using a codebook thematic analysis approach (Braun & Clarke, 2022; Brooks et al., 2015) (ATLAS.ti version 23.2.1). Using a theoretically-based initial coding framework (TFA [Sekhon et al., 2017] and multifaceted conceptualization of engagement [Cole-Lewis et al., 2019; Kelders et al., 2020; O’Brien, 2016; Perski et al., 2017; Wannheden et al., 2021]; Supplemental Appendix 4), one author inductively generated sub-codes. Categorical theming was conducted to organize the patterns constructed (Saldana, 2021). A second author independently coded transcripts, adding additional codes if needed. Independently-developed thematic frameworks (Supplemental Appendixes 5 and 6) were compared and synthesized collaboratively to generate the final interpretation. Qualitative and quantitative measures of engagement were triangulated.

## Results

### Study Flow

**Figure 1.**
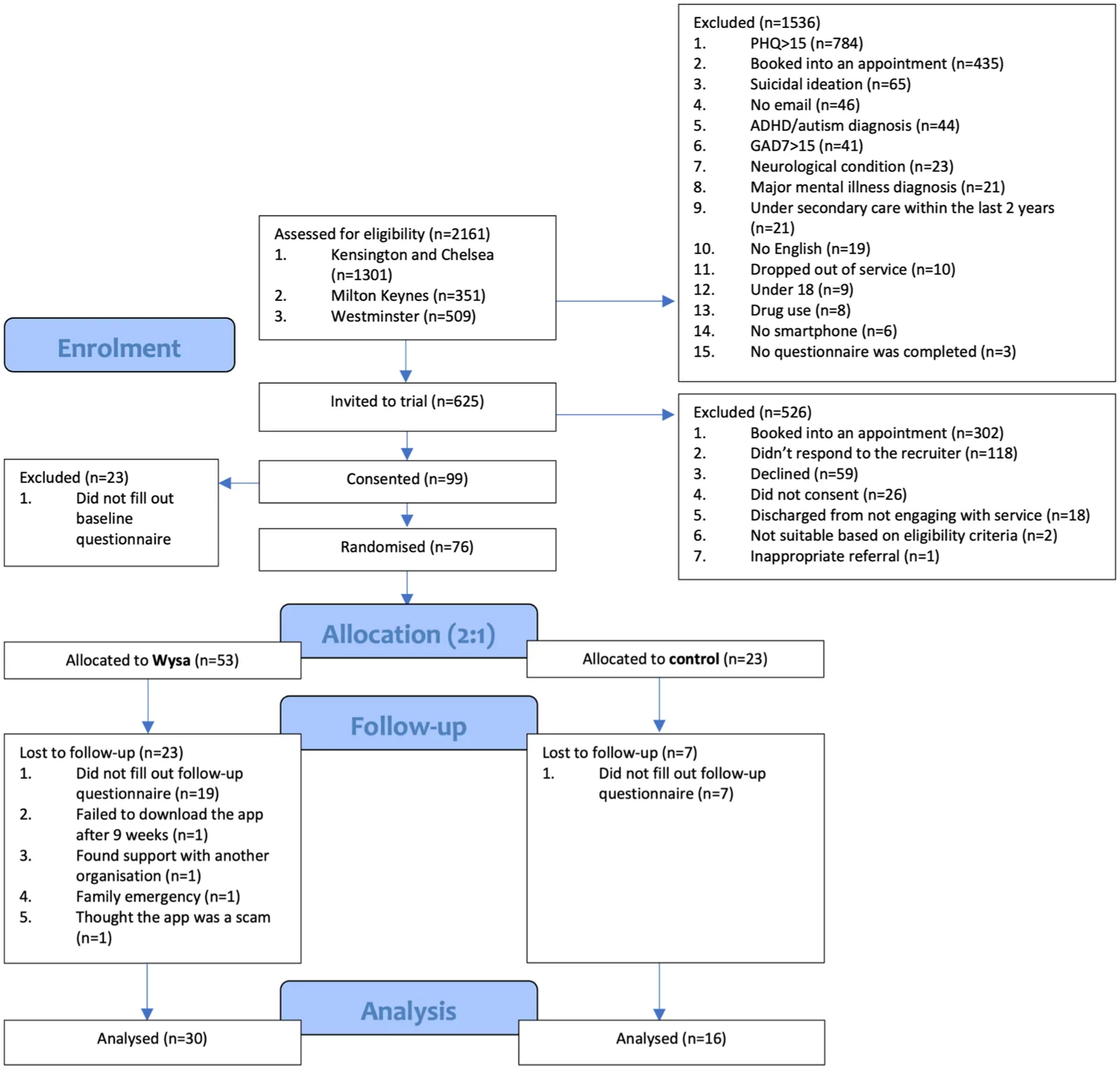
Participant flow diagram.

Two thousand one hundred and sixty one patients were assessed for eligibility; 1,536 were excluded initially and another 526 later on (Figure 1). Ninety nine consented and 76 were randomized; 46 completed follow-up and constituted the final sample. Data was collected between 13 December 2022 and 31 July 2023. For the qualitative analysis, 10 participants were reached and scheduled for interview. Six participants were “no-shows” and four completed an interview between 22 June and 25 July 2023.

### Baseline Characteristics

Baseline characteristics were similar between groups except on five variables: ethnicity, history of secondary care, HONOS cluster, domestic violence, and days on waitlist (Table 2). There were also slight imbalances for PHQ-9, GAD-7, and EQ-5D scores. Despite differences in wait time, the mean number of therapy sessions (standard care) received during the intervention period was comparable. Visualizations of variables used in regressions are provided in Supplemental Appendix 7, a data dictionary in Supplemental Appendix 8.

**Table 2.** Baseline Characteristics and Health Outcomes at Follow-Up.

Variable	Control (*N* = 16)	Wysa (*N* = 30)	Overall (*N* = 46)
Age			
Mean (*SD*)	37.1 (15.8)	36.0 (14.4)	36.3 (14.7)
Median [Min, Max]	33.0 [20.0, 77.0]	28.5 [19.0, 68.0]	31.5 [19.0, 77.0]
Gender (%)			
Female	11 (68.8)	18 (60.0)	29 (63.0)
Male	4 (25.0)	10 (33.3)	14 (30.4)
Transgender female	1 (6.3)	1 (3.3)	2 (4.3)
Not stated	0 (0)	1 (3.3)	1 (2.2)
Ethnicity (%)			
Asian or Asian British	1 (6.3)	3 (10.0)	4 (8.7)
Not known	2 (12.5)	1 (3.3)	3 (6.5)
Other ethnic groups	5 (31.3)	4 (13.3)	9 (19.6)
White	8 (50.0)	21 (70.0)	29 (63.0)
Black or Black British	0 (0)	1 (3.3)	1 (2.2)
On system 1 (history of secondary care) (%)			
No	13 (81.3)	21 (70.0)	34 (73.9)
Yes	3 (18.8)	9 (30.0)	12 (26.1)
Therapy option (%)			
Counseling	3 (18.8)	6 (20.0)	9 (19.6)
Couples counseling	1 (6.3)	0 (0)	1 (2.2)
High intensity CBT (Guided self-help)	8 (50.0)	16 (53.3)	24 (52.2)
Low intensity CBT (Guided self-help)	4 (25.0)	5 (16.7)	9 (19.6)
CfD (Counseling for depression)	0 (0)	2 (6.7)	2 (4.3)
DIT (Dynamic inter personal therapy) counseling	0 (0)	1 (3.3)	1 (2.2)
Discharged from service (%)			
No	12 (75.0)	23 (76.7)	35 (76.1)
Yes	4 (25.0)	7 (23.3)	11 (23.9)
Reason for discharge (%)			
Declined service	1 (6.3)	0 (0)	1 (2.2)
Dropped out / Treatment	1 (6.3)	2 (6.7)	3 (6.5)
Treatment completed	2 (12.5)	4 (13.3)	6 (13.0)
Declined treatment	0 (0)	1 (3.3)	1 (2.2)
Missing	12 (75.0)	23 (76.7)	35 (76.1)
HONOS cluster (%)			
Cluster 1: Common mental health problems (Low severity)	1 (6.3)	2 (6.7)	3 (6.5)
Cluster 2: Common mental health problems (Low severity, greater need)	6 (37.5)	4 (13.3)	10 (21.7)
Cluster 3: Non psychotic (Moderate severity)	9 (56.3)	16 (53.3)	25 (54.3)
Cluster 4: Non Psychotic (Severe)	0 (0)	6 (20.0)	6 (13.0)
Cluster 5: Non psychotic disorders (Very severe)	0 (0)	1 (3.3)	1 (2.2)
Not known	0 (0)	1 (3.3)	1 (2.2)
Domestic violence (%)			
No	15 (93.8)	24 (80.0)	39 (84.8)
Yes (historical)	1 (6.3)	4 (13.3)	5 (10.9)
Not known	0 (0)	2 (6.7)	2 (4.3)
Medication (%)			
Not prescribed	6 (37.5)	13 (43.3)	19 (41.3)
Prescribed but not taking	10 (62.5)	17 (56.7)	27 (58.7)
Employed: triage (%)			
No	11 (68.8)	25 (83.3)	36 (78.3)
Yes	5 (31.3)	5 (16.7)	10 (21.7)
Employed: discharge (%)			
No	13 (81.3)	27 (90.0)	40 (87.0)
Yes	3 (18.8)	3 (10.0)	6 (13.0)
Benefits (%)			
No	12 (75.0)	25 (83.3)	37 (80.4)
Not known	2 (12.5)	0 (0)	2 (4.3)
Yes	2 (12.5)	4 (13.3)	6 (13.0)
Not stated	0 (0)	1 (3.3)	1 (2.2)
Waiting list			
Mean (*SD*)	43.1 (32.2)	86.5 (52.3)	71.4 (50.5)
Median [Min, Max]	29.5 [4.0, 115]	88.5 [4.0, 208]	66.5 [4.0, 208]
Number of NHS sessions			
Mean (*SD*)	5.88 (3.36)	5.73 (3.16)	5.78 (3.20)
Median [Min, Mix]	7.00 [1.00, 11.0]	5.50 [1.00, 13.0]	6.50 [1.00, 13.0]

The 4 interviewed participants varied by gender (2 women, 1 man, 1 declined to respond), ethnicities (3 White, 1 Asian), age (3 were 25–64, 1 was 18–24), deprivation (2 each above and below median, based on postcode deprivation index), and initial PHQ-9 score (3 moderate, 1 mild).

### Clinical Outcomes

The results of the clinical outcome measures at baseline and 12 weeks are presented in Table 3.

**Table 3.** Clinical Outcome Changes Between Baseline and 12 Weeks.

Clinical outcome	Control Mean (*SD*)	Wysa Mean (*SD*)	Overall Mean (*SD*)
PHQ-9	*n* = 16	*n* = 30	*n* = 46
Baseline	11.0 (4.38)	11.9 (5.07)	11.6 (4.81)
12 weeks	8.41 (3.82)	9.28 (6.56)	8.97 (5.72)
GAD-7	*n* = 16	*n* = 30	*n* = 46
Baseline	11.9 (4.92)	11.3 (4.92)	11.5 (4.87)
12 weeks	8.19 (3.22)	8.98 (6.13)	8.71 (5.27)
EQ-5D-5L	*n* = 13	*n* = 21	*n* = 34
Baseline	0.607 (0.235)	0.681 (0.163)	0.655 (0.192)
12 weeks	0.644 (0.192)	0.688 (0.174)	0.671 (0.180)

#### Depression

**Figure 2.**
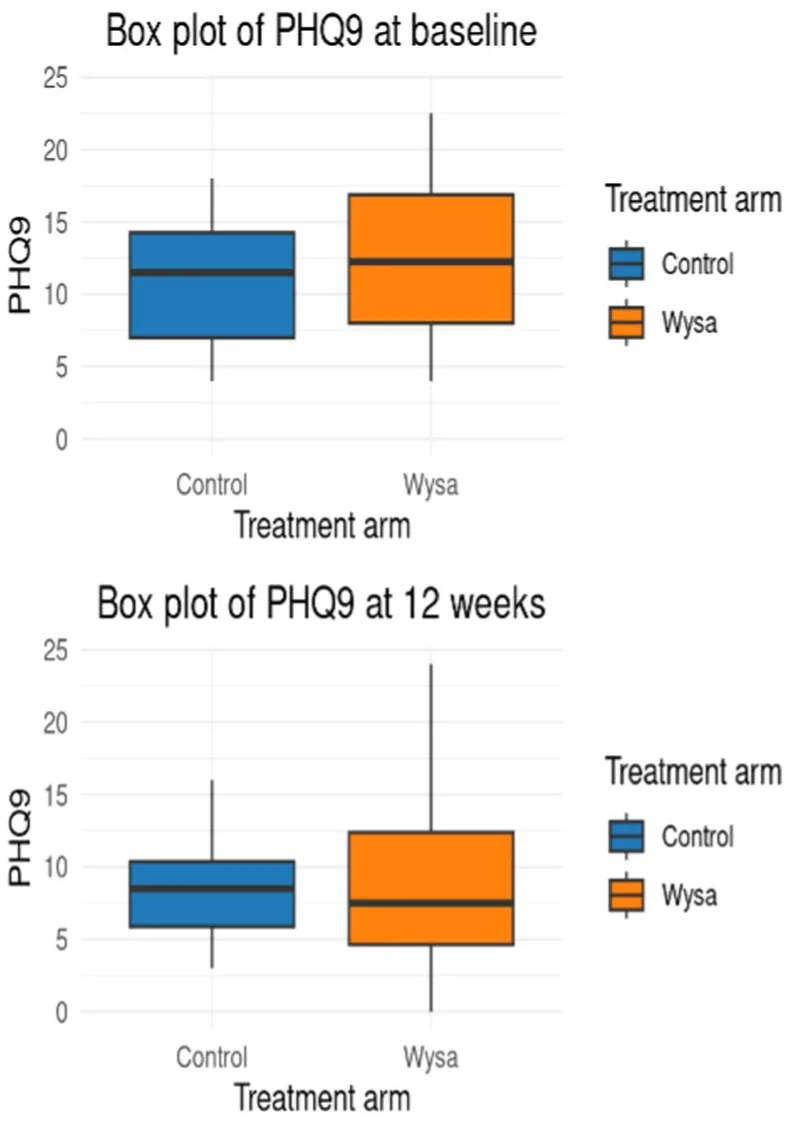
Box plots of PHQ-9 at baseline and follow-up^a^. ^a^Box plots in Figure 2 give a summary of the spread of data. The box indicates the 1^st^ and 3^rd^ quantile and the central line is the median. The whiskers indicate the minimum and maximum values and points beyond these are outliers.

For the primary outcome (PHQ-9), mean score differences between baseline and follow-up within arms were 2.62 and 2.59, for Wysa and control respectively. Standard deviations were large (5.07 at baseline, 6.56 at follow-up for Wysa; 4.38 at baseline, 3.82 at follow-up for control; Figure 2). After adjustment for baseline score the difference between PHQ-9 score was 0.2767 (95% CI [−2.8178, 3.3711]), with a wide confidence interval indicating no impact of Wysa. Multivariable linear regression indicated the potential for confounding of the unadjusted mean differences, with the coefficient of the intervention impact changing from 0.2767 (95% CI [−2.8178, 3.3711]) to 0.2160 (95% CI [−3.3079, 3.74]) after the addition of controls and number of sessions variable (Table 4), but remaining statistically insignificant. The results do not allow for inferences on the magnitude and sign of the coefficient. Due to the sample size, the coefficient may still be biased, and so it is not just the precision that is lacking, but the systematic variation of the joint distribution of patient characteristics within arms.

**Table 4.** Linear Regression Coefficients (95% Confidence Intervals, *n* = 46).

PHQ-9	Model 1	Model 2^$^	Model 3^‡^
Wysa	0.2767 (−2.8178, 3.3711)	0.2322 (−3.3293, 3.7937)	0.2160 (−3.3079, 3.74)
Control variables	No	Yes	Yes
Number of sessions	No	No	Yes

$ age, gender, ethnicity, and days on the waiting list.

‡ age, gender, ethnicity, and days on the waiting list, number of NHS therapy sessions.

As a *post-hoc* analysis, PHQ-2 scores were used as a threshold to subgroup the final sample (Supplemental Appendixes 8–9). There were small differences in coefficient magnitude but statistical significance was not reached in any model, so no additional inferences were made.

#### Anxiety

**Figure 3.**
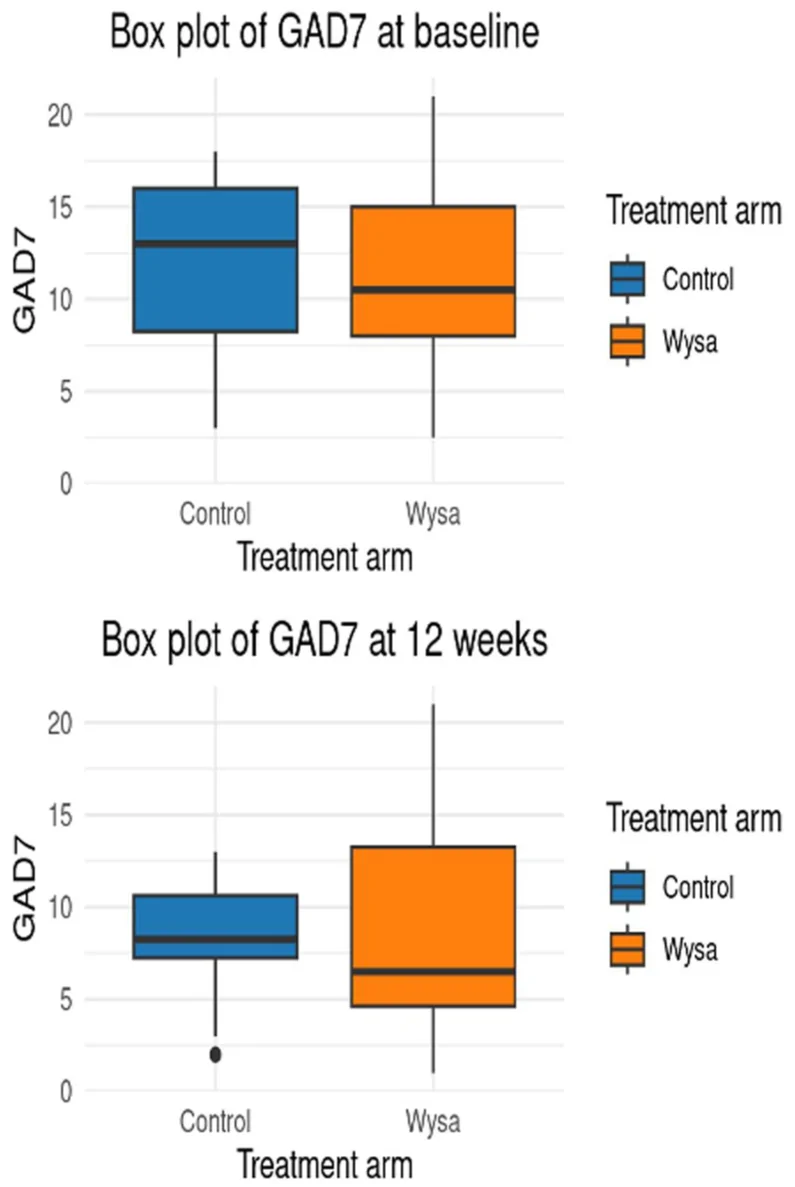
Boxplots of GAD-7 at baseline and follow-up.

Mean differences in GAD-7 scores within the arms were compared (2.32 for Wysa and 3.71 for control) but, as with PHQ-9, standard deviations were relatively large (4.92 at baseline, 6.13 at follow-up for Wysa; 4.92 at baseline, 3.22 at follow-up: for control; Figure 3). Multiple linear regression showed that the coefficient for the treatment effect variable may have been more heavily confounded than PHQ-9, as it had a larger change from 1.29 (95% CI [−1.1755, 3.7558]) to 0.7909 (95% CI [−1.6738, 3.9766]) after both controls and number of sessions were controlled for (Table 5). Similar limitations apply with regard to the inferences because of statistical insignificance and sample size.

**Table 5.** Linear Regression Coefficients (95% Confidence Intervals, *n* = 46).

GAD-7	Model 1	Model 2^$^	Model 3^‡^
WYSA	1.29 (−1.1755, 3.7558)	1.204 (−1.7154, 3.9562)	0.7907 (−1.6738, 3.9766)
Control variables	No	Yes	Yes
Number of sessions	No	No	Yes

$ age, gender, ethnicity, and days on the waiting list.

‡ age, gender, ethnicity, and days on the waiting list, number of NHS therapy sessions.

#### Health-Related Quality of Life

**Figure 4.**
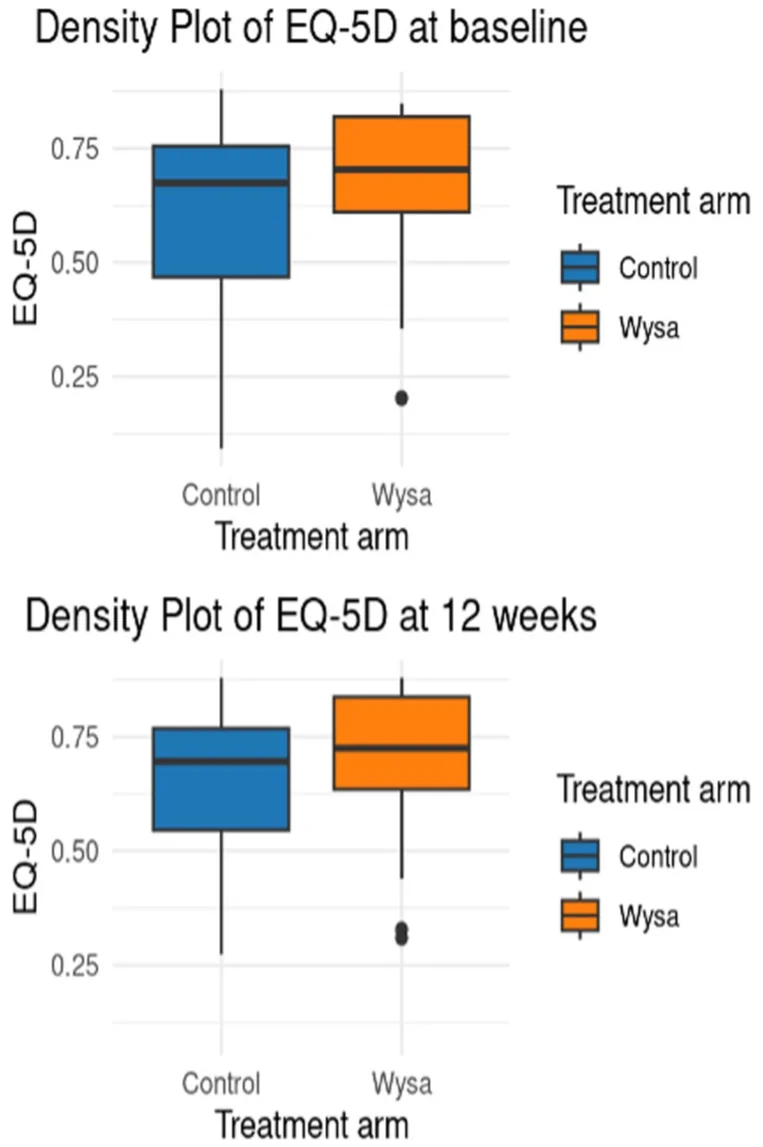
Boxplots of EQ-5D scores at baseline and follow-up.

Differences within the two arms were small for the EQ-5D-5L (0.007 in Wysa and 0.037 for control) and variation was large relative to this (Figure 4). Unlike PHQ-9 and GAD-7, variation shrunk for the control arm only. Data was collected from all participants at baseline, but there was missing data from 3 control (18.8%) and 9 intervention (30.0%) participants at 12 weeks; these participants were excluded from this analysis.

### Engagement and Acceptability

**Figure 5.**
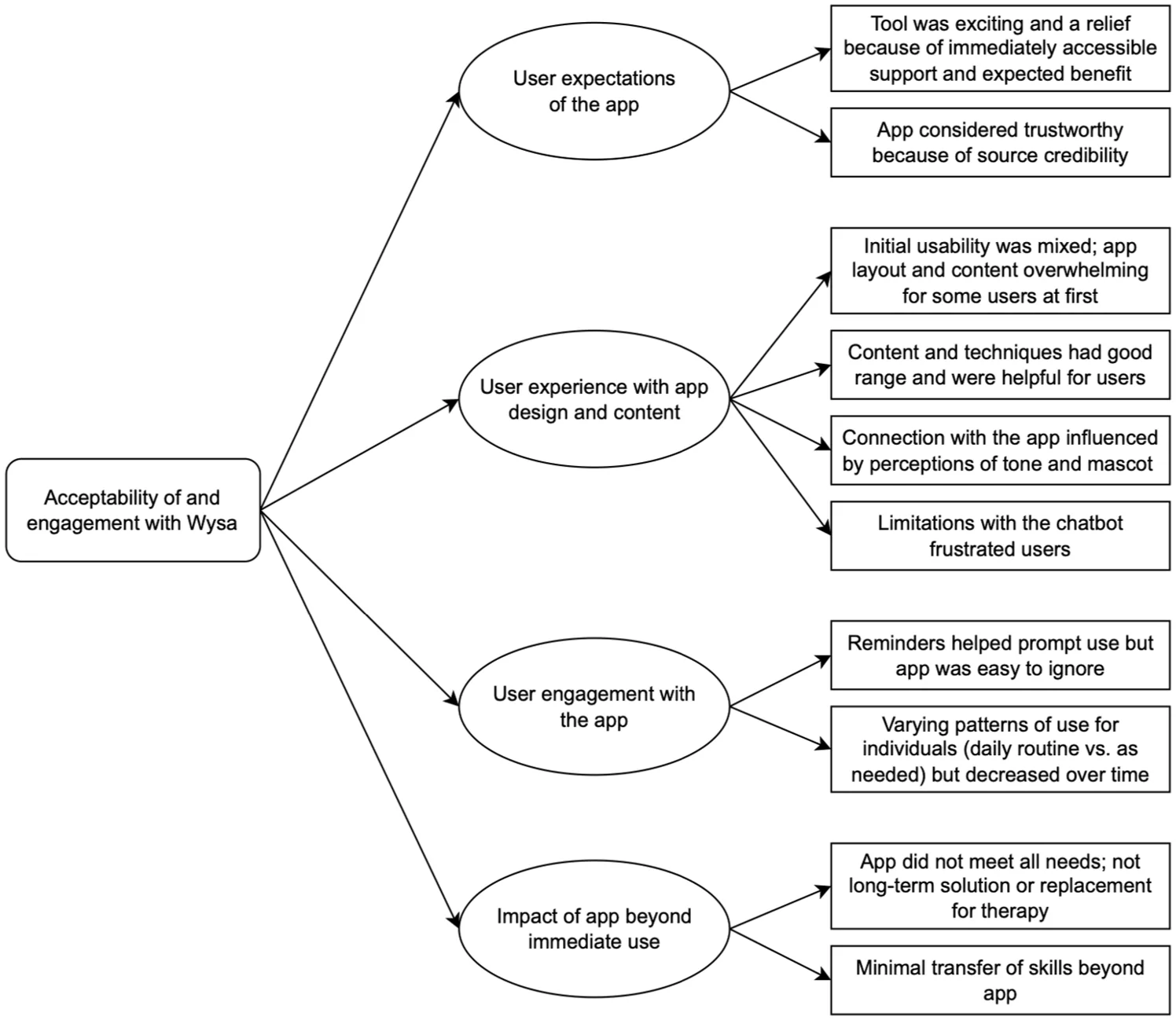
Thematic map of qualitative interviews to determine acceptability of and engagement with Wysa.

Several key themes relating to users’ experiences and engagement with the app were generated (Figure 5). Initial attitudes were positive – participants were curious, excited, and relieved to access support. Participants considered the app trustworthy because of source credibility (received via the NHS) and perceived benefits (“*credible because it helps*”). The only potential concern was privacy, but this depended on the extent of the personal details shared with the app.

The app’s tone influenced affective engagement; though perceptions differed (“*it was very judgement free*” vs. “*when you’re in that very vulnerable state, you can take it as being sarcastic or criticising*”), all highlighted the importance of a positive, genuine tone. Cognitive engagement was facilitated by ease of use – all participants found the app required little effort, although the “*amount of things you get given at the very beginning . . . was overwhelming*.” This critique was limited to the layout – the amount of activities and content available a “*highlight.*” The key issue was the CA: all four interviewees reported that the CA’s ability to converse was limited and it was predictable, repetitive, and recalled previous conversations inconsistently. This “*became frustrating*” and reduced participants’ confidence in, and use of, the app.

Patterns of app use varied. Some participants incorporated the app into daily routines, while others used it only when they had time or needed support. Participants found that the reminders helped trigger use but that the app “*was very easy to ignore.*” Some suggested enabling customization of reminders or having interesting notifications or widgets stuck to their screens to encourage them to open the app.

Participants generally felt that Wysa had some benefits (“*the chats were really helpful*”) but did not meet all of their needs (e.g. “*about 30% of the time I got what I wanted”* and “*on a scale of 10, maybe 6*”). Overall, it had potential and the resources could be “*sufficient for [some users’] needs*” but wouldn’t “*replace one-on-one therapy.*” Participants reported decreasing use of the app over time, but there was limited evidence to suggest that this was because they were incorporating skills into their daily lives.

These perspectives only represent a subset of participants (*n* = 4). Of the 30 patients who received Wysa, 40% (12/30) did not engage with it at all (Table 6).

**Table 6.** Engagement with Wysa.

Category	Over all (*N* = 30)
Engaged with Wysa	
No	12 (40.0%)
Yes	18 (60.0%)
Wysa: Exercises completed	
Mean (*SD*)	7.11 (7.79)
Median [Min, Max]	3.00 [1.00, 22.0]
Missing	21 (70.0%)
Wysa: Sessions completed	
Mean (*SD*)	24.3 (29.8)
Median [Min, Max]	11.0 [1.00, 91.0]
Missing	12 (40.0%)
Wysa: Messages to bot	
Mean (*SD*)	227 (319)
Median [Min, Max]	99.0 [20.0, 1,180]
Missing	12 (40.0%)

### Safety and Adverse Events

No adverse events were identified upon review of patients’ notes on iaptus; however, this would only capture self-reports to the service or crisis service reports. Safeguards are enabled in the system through the app’s user-triggered escalation functionality and Wysa’s automated analysis of textual language to detect potential risks – both alerts undergo human review. Within the trial, four users triggered Wysa’s escalation and the app identified and flagged suicidal language in one user. Appropriate signposting was provided for these users.

## Discussion

### Principal Findings

Previous evidence has established that mental health apps can significantly reduce symptoms of depression and anxiety compared to a control (Linardon et al., 2019; Seegan et al., 2023). Regarding the effectiveness of CAs specifically, a recent meta-analysis found a significant effect of CAs (compared to controls) on symptoms of depression (Hedges’ *g* = 0.64) (Li et al., 2023). In our study, the low sample size prevented us from determining whether Wysa had a clinically-meaningful effect on symptoms of depression and anxiety. The qualitative analysis highlighted several factors associated with engagement and acceptability. Limitations relating to CA repetitiveness caused frustration and reduced motivation to use Wysa but participants appreciated having accessible support. Further personalization of these technologies could help mitigate such limitations, which were inherent in the current version of the system.

### Limitations

Over the 4.5 month recruitment period, monthly referral rates were lower than anticipated (~800 rather than the 1,100 service target) and many patients were seen quickly and became ineligible. Had the study period been longer, this could have been neutralized by the consequence of the limitation of time for study execution. Of the ~800 monthly referrals, ~530 patients (65%) were screened and ~100 (12.5%) met eligibility criteria. Removing PHQ-9 and GAD-7 eligibility cut-offs, adding incentives, and adding sites were considered to increase recruitment. As PHQ-9 and GAD-7 are for screening (not diagnosis), removing cut-offs could have changed the focus from mild-moderate to moderate-severe symptoms and increased patient risk. Incentives have been associated with lower attrition (Linardon & Fuller-Tyszkiewicz, 2020; Treweek et al., 2018) but their evidence in mental health research is mixed and risked exploiting a vulnerable population (Borschmann et al., 2014; Y. Liu et al., 2018; Resnik, 2015). We were unable to include additional Trusts or extend the study due to funding restrictions. Including additional Trusts, particularly those with longer waiting lists, may have increased study uptake. A pragmatic analytic approach was taken to inform future trial design.

Due to the low recruitment numbers, it is not clear whether our study population is representative of the larger surrounding population. This includes characteristics of social deprivation, age profile, education and tech awareness.

Another limitation was our inability to complete all of the planned analyses within the time allotted for the study by the funder. Due to delays with ethical and regulatory approvals, we were unable to collect all planned data: cost analysis data, a second measure of health-related quality of life (the Short Form 12 [SF-12] health survey [SF-12v2® Health Survey, 2021]), Emergency Department (A&E) or CNWL crisis helpline use, or in-app acceptability reviews.

### Meaning in the Context of the Literature

The study generated useful insights into how and why users engaged with a mental health CA. This was a key aspect because lack of engagement is a common challenge for digital health interventions (Baumel et al., 2019; Birnbaum et al., 2015; Meyerowitz-Katz et al., 2020; Pratap et al., 2020; Torous et al., 2020; Yeager & Benight, 2018) and engagement has been positively associated with improved mental health (Daley et al., 2020; Inkster et al., 2018). Understanding how and why participants engaged with AI intervention is critical: in the context of AI-driven tools, where user trust, perceived credibility, and the ability to personalize or adapt to user needs can strongly influence uptake, interpreting engagement goes beyond simple usage metrics; it also provides insight into potential barriers and enablers of therapeutic effectiveness. Our finding that 40% of participants (12/30) did not engage at all was in line with a recent meta-analysis of smartphone-based mental health interventions that found that a mean of 41% of participants failed to download interventions targeting depression (Linardon & Fuller-Tyszkiewicz, 2020). Qualitative findings were also aligned with previous evidence of mixed opinions about chatbots in healthcare (Young et al., 2021) and that repetition and misunderstandings by CAs are annoying and reduce motivation to engage (Boucher et al., 2021). Addressing limitations to reduce repetitiveness and make it more anthropomorphic could cause serious ethical and safety issues. There are preliminary reports that generative AI can cause harm by producing unpredictable responses, presenting misinformation, crossing the line between being a “professional” versus a “friend,” or unintentionally motivating harmful thoughts and actions (AI and Eating Disorders: How Generative AI Is Enabling Users to Generate Harmful Eating Disorder Content, 2023; De Freitas et al., 2023). Rule-based AI, while limiting conversation complexity, also restricts the CA to providing clinically pre-approved responses.

### Further Studies

The study also highlighted key lessons for future study design:

- Ensure a clear understanding of service demand; up-to-date and long-term data (past 12 months) should be collected from interested sites to determine which, and how many, sites are needed to achieve recruitment targets.
- Include sites with sufficient eligible participants to greatly exceed the recruitment target; attrition rates for digital health interventions can be up to 50% (Linardon & Fuller-Tyszkiewicz, 2020; Meyerowitz-Katz et al., 2020; Torous et al., 2020).
- Tool deployment and eligibility considerations should be discussed with service providers but ultimately the clinical chief investigator must ensure minimal risk.
- Plan for a long study period; intervention periods should account for the possibility of approval and set-up delays.
- Set expectations with funding bodies at the beginning and discuss contingency plans and the possibility for extensions if study delivery issues arise.

## Conclusions

The study demonstrated potential for a mobile CA to provide mental health support to supplement standard treatment. It appears Wysa is a supportive intervention and developing this to support waiting list has potential. However, it cannot yet be considered as an alternative to standard psychological therapies. This would require further evidence.

Participants expressed relief to have a source of support while waiting for services and found its content useful. Limitations with the CA’s ability to hold in-depth conversations frustrated users and negatively influenced engagement. Study execution challenges highlighted the difficulties of proving effectiveness for improving mental health outcomes. Despite conducting the traditional “gold standard” RCT design, we identified several factors influencing successful study execution. Lessons learned included the need to verify recruitment estimates to ensure sufficient demand and to build in contingency to accommodate unexpected delays and higher-than-anticipated ineligibility or attrition. The issues experienced suggest that an RCT may not be the ideal study design for assessing digital health interventions, which require strong evidence from large populations in a timely manner to keep up with their development and implementation in healthcare. This is a point of discussion within the digital health field that deserves further consideration (Guo et al., 2020; Hrynyschyn et al., 2022; Pham et al., 2016).

## Supplemental Material

sj-docx-1-isp-10.1177_00207640251415507 – Supplemental material for Real-World Testing of an Artificial Intelligence Conversational Agent as an Early Intervention and Support Tool in the Mental Health Referral Care Pathway: A Mixed-Methods Study Supplemental material, sj-docx-1-isp-10.1177_00207640251415507 for Real-World Testing of an Artificial Intelligence Conversational Agent as an Early Intervention and Support Tool in the Mental Health Referral Care Pathway: A Mixed-Methods Study by Edward Meinert, Madison Milne-Ives, Emma Taylor, Becky Inkster, Alina Paik, Ananya Ananthakrishnan, Martin Orr, Ceire Costelloe and Rohit Shankar in International Journal of Social Psychiatry
