# Supplementary material for: Real-World Testing of an Artificial Intelligence Conversational Agent as an Early Intervention and Support Tool in the Mental Health Referral Care Pathway: A Mixed-Methods Study
Source: Int J Soc Psychiatry. 2026 Mar 5;72(6):1819–32. doi: 10.1177/00207640251415507 (PMC13569919; doi:10.1177/00207640251415507)
Supplement: Supplementary file 1 [file sj-docx-1-isp-10.1177_00207640251415507.docx]

# Appendices

## Appendix 1. Wisconsin logic diagram [(Taylor-Powell & Henert, 2008)](https://paperpile.com/c/0UufJ0/eQEAr)

## Appendix 2. CONSORT-AI Checklist [(X. Liu et al., 2020)](https://paperpile.com/c/0UufJ0/l4jcX)

| Section | Item | CONSORT 2010 Item^a^ | CONSORT-AI Item | | Page No |
| --- | --- | --- | --- | --- | --- |
| Title and Abstract | | | | | |
| **Title and Abstract** | 1a | Identification as a randomised trial in the title | CONSORT-AI 1a,b Elaboration | (i) Indicate that the intervention involves artificial intelligence/machine learning in the title and/or abstract and specify the type of model. | Title page |
|  |  |  |  |  |  |
|  | 1b | Structured summary of trial design, methods, results, and conclusions (for specific guidance see CONSORT for abstracts) |  | (ii) State the intended use of the AI intervention within the trial in the title and/or abstract. | Title, abstract |
|  |  |  |  |  |  |
| Introduction | | | | | |
| **Background and objectives** | 2a | Scientific background and explanation of rationale | CONSORT-AI 2a (i) Extension | Explain the intended use of the AI intervention in the context of the clinical pathway, including its purpose and its intended users (e.g. healthcare professionals, patients, public). | 3 |
|  |  |  |  |  |  |
|  | 2b | Specific objectives or hypotheses |  |  | 3 |
| Methods | | | | | |
| **Trial design** | 3a | Description of trial design (such as parallel, factorial) including allocation ratio |  |  | 3 |
|  | 3b | Important changes to methods after trial commencement (such as eligibility criteria), with reasons |  |  | 5-6 |
| **Participants** | 4a | Eligibility criteria for participants | CONSORT-AI 4a (i) Elaboration | State the inclusion and exclusion criteria at the level of participants. | 4 (Table 1) |
|  |  |  | CONSORT-AI 4a (ii) Extension | State the inclusion and exclusion criteria at the level of the input data. | N/A |
|  | 4b | Settings and locations where the data were collected | CONSORT-AI 4b Extension | Describe how the AI intervention was integrated into the trial setting, including any onsite or offsite requirements. | 4-5 |
| **Interventions** | 5 | The interventions for each group with sufficient details to allow replication, including how and when they were actually administered | CONSORT-AI 5 (i) Extension | State which version of the AI algorithm was used. | 4 |
|  |  |  | CONSORT-AI 5 (ii) Extension | Describe how the input data were acquired and selected for the AI intervention. | 4-5 |
|  |  |  | CONSORT-AI 5 (iii) Extension | Describe how poor quality or unavailable input data were assessed and handled. | N/A |
|  |  |  | CONSORT-AI 5 (iv) Extension. | Specify whether there was human-AI interaction in the handling of the input data, and what level of expertise was required of users. | 4-5 |
|  |  |  | CONSORT-AI 5 (v) Extension | Specify the output of the AI intervention | 4-5 |
|  |  |  | CONSORT-AI 5 (vi) Extension | Explain how the AI intervention’s outputs contributed to decision-making or other elements of clinical practice. | N/A |
| **Outcomes** | 6a | Completely defined pre-specified primary and secondary outcome measures, including how and when they were assessed |  |  | 5 |
|  | 6b | Any changes to trial outcomes after the trial commenced, with reasons |  |  | 15 |
| **Sample size** | 7a | How sample size was determined |  |  | 5 |
|  | 7b | When applicable, explanation of any interim analyses and stopping guidelines |  |  | N/A |
| Randomisation | | | | | |
| **Sequence generation** | 8a | Method used to generate the random allocation sequence |  |  | 4 |
|  | 8b | Type of randomisation; details of any restriction (such as blocking and block size) |  |  | 3-4 |
| **Allocation concealment mechanism** | 9 | Mechanism used to implement the random allocation sequence (such as sequentially numbered containers), describing any steps taken to conceal the sequence until interventions were assigned |  |  | 4 |
| **Implementation** | 10 | Who generated the random allocation sequence, who enrolled participants, and who assigned participants to interventions |  |  | 4 |
| **Blinding** | 11a | If done, who was blinded after assignment to interventions (for example, participants, care providers, those assessing outcomes) and how |  |  | 4 |
|  | 11b | If relevant, description of the similarity of interventions |  |  | N/A |
| **Statistical methods** | 12a | Statistical methods used to compare groups for primary and secondary outcomes |  |  | 5-6 |
|  | 12b | Methods for additional analyses, such as subgroup analyses and adjusted analyses |  |  | 5-6 |
| Results | | | | | |
| **Participant flow** (a diagram is strongly recommended) | 13a | For each group, the numbers of participants who were randomly assigned, received intended treatment, and were analysed for the primary outcome |  |  | 6, Figure 1 |
|  | 13b | For each group, losses and exclusions after randomisation, together with reasons |  |  | Figure 1 |
| **Recruitment** | 14a | Dates defining the periods of recruitment and follow-up |  |  | 3, 5 |
|  | 14b | Why the trial ended or was stopped |  |  | 15 |
| **Baseline data** | 15 | A table showing baseline demographic and clinical characteristics for each group |  |  | 7, Table 2 |
| **Numbers analysed** | 16 | For each group, number of participants (denominator) included in each analysis and whether the analysis was by original assigned groups |  |  | Table 2 |
| **Outcomes and estimation** | 17a | For each primary and secondary outcome, results for each group, and the estimated effect size and its precision (such as 95% confidence interval) |  |  | 9-12 |
|  | 17b | For binary outcomes, presentation of both absolute and relative effect sizes is recommended |  |  | N/A |
| **Ancillary analyses** | 18 | Results of any other analyses performed, including subgroup analyses and adjusted analyses, distinguishing pre-specified from exploratory |  |  | 12-14 |
| **Harms** | 19 | All important harms or unintended effects in each group (for specific guidance see CONSORT for harms) | CONSORT-AI 19 Extension | Describe results of any analysis of performance errors and how errors were identified, where applicable. If no such analysis was planned or done, explain why not. | 14, N/A |
| Discussion | | | | | |
| **Limitations** | 20 | Trial limitations, addressing sources of potential bias, imprecision, and, if relevant, multiplicity of analyses |  |  | 15 |
| **Generalisability** | 21 | Generalisability (external validity, applicability) of the trial findings |  |  | 15-16 |
| **Interpretation** | 22 | Interpretation consistent with results, balancing benefits and harms, and considering other relevant evidence |  |  | 14-16 |
| Other Information | | | | | |
| **Registration** | 23 | Registration number and name of trial registry |  |  | 1 |
| **Protocol** | 24 | Where the full trial protocol can be accessed, if available |  |  | N/A |
| **Funding** | 25 | Sources of funding and other support (such as supply of drugs), role of funders | CONSORT-AI 25 Extension. | State whether and how the AI intervention and/or its code can be accessed, including any restrictions to access or re-use. | 17 |

## Appendix 3. SSI topic guide

**Introduction**

Hello ___, it’s nice to meet you. I really appreciate you taking the time to be involved in this evaluation, it will be really helpful. My name is Madison Milne-Ives and I am a researcher at the Centre for Health Technology at the University of Plymouth who is working on the Wysa evaluation project.

Purpose of our investigation is to evaluate the Wysa app - we weren’t involved in development, we just want to figure out if it would be a useful tool to bring into the NHS mental health service provision in addition to standard care. Your feedback is very valuable for us.

I just want to remind you that all of your answers will be kept confidential and stored on a password-protected computer that only we can access. My questions are about your experience using the Wysa app, not about your mental health, but if there are any questions you don’t want to answer, that’s completely fine, just let me know and we’ll move on. We can also stop at any point you want and you don’t need to give us a reason.

Before we start, is it okay with you if I audio-record this session?

- The reason we record is so we can go back and remember what was said and what wasn’t said. The audio recording that we’re taking today will be deleted as soon as it has been transcribed, and any identifying information (like your name) will be removed.
- NOTE: ask again for confirmation once recording is on
- If no: “That’s fine, is it okay if I type notes as we talk and share those notes with you once we have finished the interview so you can make sure they are accurate?”

Today we’re going to be going through some questions to discuss your experiences using the app. If there is anything you don’t understand during the interview, feel free to ask!

Before we get started, do you have any questions?

**Questions**

1. Thinking back to when you first got the app, can you tell me about what you felt and thought about using it? (*TFA - affective attitude, engagement - affective, cognitive*)
2. What motivated you to use the app? Why did you want to try it? *(engagement - affective)*
   a. What did you hope to gain / how did you think it would benefit you? *(TFA - perceived effectiveness, anticipated)*
3. How much effort did it take to use Wysa? What made it easy or difficult to use? *(TFA - burden)*
   a. How confident were you in your ability to use the app? *(TFA - self-efficacy)*
   b. How well did you understand how Wysa worked? *(TFA - intervention coherence)*
4. How did using the app - or specifically the general talk feature or the self-care exercise packs - make you feel? *(engagement - micro, affective)*
   a. What aspects of the app did you find most and least enjoyable? Why?
5. What did you think about the app’s chat function and exercise pack content? *(engagement - micro, cognitive)*
   a. What did you find most interesting / attention catching? Why?
   b. What was most boring? Why?
   c. What affected your interest or the attention you paid to the app?
6. Did you find the program and resources credible and trustworthy? What elements of the program fostered that trust/distrust for you? (*engagement - affective (social trust), cognitive*)
7. What concerns, if any, did you have about using Wysa? *(TFA - ethicality)*
   a. To what degree did the app’s features and content align with your values and thoughts about mental health support?
8. How much confidence did you have in Wysa’s ability to provide mental health support? *(TFA - perceived effectiveness)*
9. What, if anything, did you have to give up to engage with Wysa? Were there any costs or drawbacks to using the app? *(TFA - opportunity costs)*
10. Do you think the intervention influenced you beyond when you were using the app? If so, how? (*engagement - behavioural, TFA - perceived effectiveness*)
    a. For example, having more skills and strategies or feeling more capable of managing stress, sleeping better, etc.
11. Can you describe how you incorporated the app into your daily life over the past 3 months? *(engagement - micro, behavioural)*
    a. How did you use the app? What features did you use the most / least? Why?
12. How did your use of the app change over time? (*engagement - micro)*
    a. In what circumstances were you more likely to engage with the app? Ie. Why did you log on when you did? *(engagement - micro)*
    b. What made you want to open up the app again?
    c. Why did you continue to use the app or discontinue use of the app?
    d. If you stopped using the app, why would you / did you start using it again?
13. Do you have any other feedback you’d like to provide that you don’t feel like we have discussed?

Thank you very much for taking the time to participate, it’s been really helpful to hear more about your experience and we really appreciate it. Do you have any questions for me at this stage?

## Appendix 4. Initial codebook used in preliminary qualitative coding

| **Code** | **Definition** |
| --- | --- |
| *Engagement components* | |
| Affective engagement | Motivation, positive and negative feelings (e.g. enjoyment), sensory pleasure of UX [(Kelders et al., 2020b; O’Brien, 2016; Perski et al., 2017b)](https://paperpile.com/c/0UufJ0/vtbz5+ioynj+dsJHN) |
| Cognitive engagement | Challenge, interest, thought, attention [(Kelders et al., 2020b; O’Brien, 2016; Perski et al., 2017b)](https://paperpile.com/c/0UufJ0/vtbz5+ioynj+dsJHN) |
| Behavioural engagement | Interactivity, usage (amount, duration, frequency, etc.) [(Kelders et al., 2020b; O’Brien, 2016; Perski et al., 2017b)](https://paperpile.com/c/0UufJ0/vtbz5+ioynj+dsJHN) |
| Micro engagement | Engagement with the mobile health app itself (UX / BCTs) [(Yardley et al., 2016)](https://paperpile.com/c/0UufJ0/2T9WG) |
| Macro engagement | “Engagement and identification with the wider intervention goals” [(Yardley et al., 2016)](https://paperpile.com/c/0UufJ0/2T9WG), engagement with the targeted health behaviour [(Cole-Lewis et al., 2019b)](https://paperpile.com/c/0UufJ0/oub7k) |
| *Acceptability constructs (TFA)* | |
| Affective attitude | How an individual feels about the intervention |
| Burden | The perceived amount of effort that is required to participate in the intervention |
| Ethicality | The extent to which the intervention has good fit with an individual's value system |
| Intervention coherence | The extent to which the participant understands the intervention and how it works |
| Opportunity costs | The extent to which benefits, profits, or values must be given up to engage in the intervention |
| Perceived effectiveness | The extent to which the intervention is perceived to be likely to achieve its purpose |
| Self-efficacy | The participant's confidence that they can perform the behaviour (s) required to participate in the intervention |
| Other | Utterances that answer the research question but do not necessarily reflect the TFA constructs |

## Appendix 5. Independently developed thematic framework 1

| **Engagement component** | **TFA construct** | **Code (*sub-codes*)** | | **Theme** | **Organising theme** |
| --- | --- | --- | --- | --- | --- |
| Affective | Affective attitudes | Affective attitudes - anticipated | | Tool was exciting and a relief because of accessible support and expected benefit | Factors associated with engagement |
| Affective | Affective attitudes |  | *Curious about trial / new tool* |  |  |
| Affective | Affective attitudes |  | *Excited about using* |  |  |
| Affective | Affective attitudes |  | *Worried about lack of support on wait list* |  |  |
| Affective | Affective attitudes | Relief | |  |  |
| Affective | Affective attitudes | Liked accessibility | |  |  |
| Affective | Affective attitudes, Ethicality | Accessing help without bothering anyone | |  |  |
| Affective | Affective attitudes | No reluctance | |  |  |
| Cognitive | Perceived effectiveness | Perceived effectiveness - anticipated | |  |  |
| Cognitive | Perceived effectiveness |  | *Expected emotional benefits / guidance* |  |  |
| Cognitive | Perceived effectiveness |  | *High expectations of conversational agent* |  |  |
| Cognitive | Perceived effectiveness |  | *No particular expectations of ability - not expecting the world* |  |  |
| Affective | Affective attitudes | Tone | | Tone affected user connection with app |  |
| Affective | Affective attitudes |  | *Positive, genuine, non-judgmental* |  |  |
| Affective | Affective attitudes |  | *Tone could be misinterpreted as condescending* |  |  |
| Affective | Affective attitudes | Liked mascot (approachable) | |  |  |
| Affective | Affective attitudes | Not convinced about mascot | |  |  |
| Affective | Affective attitudes | Frustration with conversational agent | | conversational agent limitations frustrated users |  |
| Affective | Affective attitudes | Lack of personal connection | |  |  |
| Affective | Affective attitudes | Tedious | |  |  |
| Cognitive | Burden | Made inputs simple for conversational agent | |  |  |
| Cognitive | Perceived effectiveness | Perceived effectiveness - experienced | |  |  |
| Cognitive | Perceived effectiveness |  | *Different from discussing with a person* |  |  |
| Cognitive | Perceived effectiveness |  | *Confidence decreased with conversational agent* |  |  |
| Cognitive | Perceived effectiveness |  | *Inconsistencies from app / conversational agent doesn't listen* |  |  |
| Cognitive | Perceived effectiveness |  | *Limited / predictable / repetitive algorithm* |  |  |
| Behavioural | - | Micro engagement | |  |  |
| Behavioural | - |  | *Issues/frustrations with conversational agent affected use* |  |  |
| Affective | Ethicality | Privacy concerns associated with what's shared with app | | Trustworthiness shaped by source credibility and personal experience |  |
| Affective | Ethicality | No concerns | |  |  |
| Affective, Cognitive | - | Trustworthy | |  |  |
| Affective, Cognitive | - | Trusted because NHS / sources cited | |  |  |
| Cognitive | Perceived effectiveness |  | *Experience of it helping improves credibility* |  |  |
| Cognitive | Burden | A bit overwhelming | | Easy to use but a bit overwhelming |  |
| Cognitive | Burden | Easy to use | |  |  |
| Cognitive | Self-efficacy | Confident in ability to use | |  |  |
| Cognitive | Opportunity costs | No costs | |  |  |
| Cognitive | Opportunity costs | Short videos with timings were good | |  |  |
| Cognitive | - | Categorisation might hide useful content | |  |  |
| Behavioural | - | Micro engagement | | Reminders helped prompt use but app was easy to ignore |  |
| Behavioural, Cognitive | - |  | *Easy to ignore / nothing grabs attention* |  |  |
| Behavioural, Cognitive | - |  | *Reminder prompted use* |  |  |
| Behavioural, Cognitive | - |  | *Tailoring reminder times would be good* |  |  |
| Affective | Affective attitudes | No incentive (esp. if emotional) | |  |  |
| Cognitive | - | App suggestions helpful - keep fresh | | Suggestions for improvement |  |
| Cognitive | - | Create a personal library | |  |  |
| Cognitive | Burden |  | *Video not always convenient - other suggestions* |  |  |
| Behavioural | - | Macro engagement | |  |  |
| Behavioural, Cognitive | - |  | *Want more training for using skills outside app* |  |  |
| Cognitive | Perceived effectiveness | Perceived effectiveness - experienced | | Range of resources were useful for users | Perceptions of potential usefulness in clinical pathways |
| Cognitive | Perceived effectiveness |  | *Confident in ability to support* |  |  |
| Cognitive | Perceived effectiveness |  | *Having something to talk to was good* |  |  |
| Cognitive | Perceived effectiveness |  | *Good range of activities* |  |  |
| Cognitive | Perceived effectiveness |  | *Liked breathing aspect* |  |  |
| Cognitive | Perceived effectiveness |  | *Resources were helpful (at least short-term/for some)* |  |  |
| Cognitive | Perceived effectiveness |  | *Sleep benefits* |  |  |
| Cognitive | Perceived effectiveness |  | *Chats helped* |  |  |
| Cognitive | - | conversational agent helped reflection | |  |  |
| Cognitive, Behavioural | - | Helped prompt previously learned techniques | |  |  |
| Affective | Affective attitudes | Liked videos | |  |  |
| Cognitive | Perceived effectiveness | Perceived effectiveness - experienced | | App did not meet all needs; not long-term solution or replacement for therapy |  |
| Cognitive | Perceived effectiveness |  | *Benefits depend on user not just tool (e.g. motivation, experience with therapy, mental health issues)* |  |  |
| Cognitive | Perceived effectiveness |  | *Not effective* |  |  |
| Cognitive | Perceived effectiveness |  | *Not for long-term / replacing therapy* |  |  |
| Cognitive | Perceived effectiveness |  | *Premium better than free?* |  |  |
| Cognitive | Perceived effectiveness |  | *Got some of what they wanted out of the app* |  |  |
| Cognitive | - | Want to understand issues | |  |  |
| Behavioural | - | Micro engagement | | Varying patterns of use for individuals | How patients used the app |
| Behavioural, Cognitive | Burden, Opportunity costs |  | *Don't always have time* |  |  |
| Behavioural | - |  | *Not using regularly* |  |  |
| Behavioural | - |  | *Used in quiet moments of day* |  |  |
| Behavioural | - |  | *Used to satisfy immediate need* |  |  |
| Behavioural | - |  | *Built into daily routine* |  |  |
| Behavioural | - |  | *Lifestyle and other factors could affect use* |  |  |
| Behavioural | - |  | *Didn't use all features* |  |  |
| Behavioural | - | Micro engagement | | Decrease in use over time |  |
| Behavioural | - |  | *Decrease in use over time* |  |  |
| Behavioural | - | Macro engagement | | Minimal transfer of skills beyond app |  |
| Behavioural | - |  | *Not much outside app* |  |  |
| Behavioural | - |  | *Once you have what you need, don't need app* |  |  |
| Behavioural | - |  | *Used skills beyond app* |  |  |

## Appendix 6. Independently developed thematic framework 2

| **Themes** | **Codes** |
| --- | --- |
| Feelings about app before use | - Curious about new tool - Excited about using - Worried about lack of support on wait list - Confident in ability to use - Accessing help without bothering anyone - Want to understand issues - Relief - Experience with other conversational agents (fed into expectations) |
| Expectations of app | - Expected emotional benefits/guidance - High expectations of conversational agent - No particular expectations of ability/not expecting the world |
| Trustworthiness/credibility of app | - Trustworthy - Trusted because NHS / sources cited - Experience of it helping improves credibility |
| Ease of use | - Easy to use - Gets easier to use over time - Made inputs simple for conversational agent - A bit overwhelming |
| User Interface (UI) | - Took time to get used to UI - Layout was overwhelming (UI) - Would like customisability (UI) - Categorisation might hide useful content |
| Engagement – positives | - Built into daily routine - Reminder prompted use - Used to satisfy immediate need - Used in quiet moments of day - No reluctance |
| Issues with engagement | - Don’t always have time - Easy to ignore/nothing grabs attention - Issues/frustrations with conversational agent affected use - Lifestyle and other factors could affect use - Tailoring reminder times would be good - Video not always convenient – other suggestions - Decrease in use over time - No incentive (esp. if emotional) - Not using regularly - Didn’t use all features |
| Positive aspects of the app | - conversational agent helped reflection - Create a personal library - Helped prompt previously learned techniques - Liked mascot (approachable) - Liked videos - Used skills beyond app - Chats helped - Confident in ability to support - Good range of activities (different means to talk about experience) - Got some of what they wanted out of the app - Having something to talk to was good - Liked breathing aspect - Liked meditation techniques - Resources were helpful (at least short-term/for some) - Sleep benefits - Short videos with timings were good - Liked accessibility |
| Concerns/issues with the app | - Frustration with conversational agent - Once you have what you need, don’t need app - Want more training for using skills outside app - Not convinced about mascot - Other conversational agent was better - Confidence decreased with conversational agent - Inconsistencies from app/conversational agent doesn’t listen - Not effective - Not enough depth - Not for long-term/replacing therapy - Satisfied immediate need but no retention - Tedious - Limited/predictable/repetitive algorithm |
| Tone/Inclusivity | - Tone - Positive, genuine, non-judgemental - Tone could be misinterpreted as condescending - Improve inclusivity with tone indicators |
| Human aspect | - Lack of personal connection - Different from discussing with a person |
| Privacy concerns | - No concerns |
| Other | - No costs (in context of – no costs to using the app) - Premium better than free? (a participant wondered) - Setting expectations would be good (what the app can and can’t do) - Benefits depend on user not just tool (e.g. motivation, experience with therapy, mental health issues) – three participants said but what category? |

## Appendix 7. Demographic visualisations

Appendix 7a. Boxplots of age, days on the waiting list and number of NHS sessions

Appendix 7b. Paired bar chart of ethnicity and gender

## Appendix 8. Data dictionary

| **Variable name** | **Description** |
| --- | --- |
| Age | Age of patient at triage |
| Gender | Gender of patient at triage |
| Ethnicity | Ethnicity of patient |
| On System 1 (history of secondary care) | Indicator whether a patient was on system 1 as having a history of secondary care |
| Therapy option | Therapy the patient is on the waiting list to receive |
| Discharge from service | Indicator whether a patient was discharged from the NHS |
| HONOS cluster | HONOS cluster the patient is classed as |
| Domestic violence | Indicator whether a patient was reported as experiencing domestic violence currently or historically |
| Medication | Prescription and adherence to psychotropic medication |
| Employed: triage | Indicator whether a patient was employed or not at triage |
| Employed: discharge | Indicator whether a patient was employed or not when they are discharged from the NHS |
| Benefits | Indicator whether the patient is receiving benefits |
| Waiting list | Number of days the patient has spent on the waiting list |
| Number of NHS sessions | Number of sessions the patient has had with the NHS for their mental health |
| EQ-5D at baseline | EQ-5D index, calculated through the R package ‘eq5d’ from an EQ-5D survey administered at baseline |
| EQ-5D at 12 weeks | EQ-5D index, calculated through the R package ‘eq5d’ from an EQ-5D survey administered at follow-up |
| PHQ-9 at baseline | An average score between the different source of PHQ-9 data at baseline: an average over the NHS survey and Wysa (if applicable) |
| PHQ-9 at 12 weeks | An average score between the different source of PHQ-9 data at follow-up: an average over the NHS survey and Wysa (if applicable) |
| GAD-7 at baseline | An average score between the different source of GAD-7 data at baseline: an average over the NHS survey and Wysa (if applicable) |
| GAD-7 at 12 weeks | An average score between the different source of GAD-7 data at follow-up: an average over the NHS survey and Wysa (if applicable) |
| Wysa | A variable to indicate whether the patient was randomised to Wysa |
| Controls | A group of variables including: age, gender, ethnicity and days on the waiting list) |

## Appendix 9. Regression coefficients (95% confidence intervals) of post hoc analysis results with PHQ2 scores of 5 and less

| PHQ-9 | Model 1 | Model 2 | Model 3 |
| --- | --- | --- | --- |
| WYSA | 0.0994  (-3.006, 3.205) | -0.5395  (-3.66, 3.3539) | -0.1517  (-3.5968, 3.2934) |
| Control variables | No | Yes | Yes |
| Number of sessions | No | No | Yes |

N = 43

## Appendix 10. Regression coefficients (95% confidence intervals) of post hoc analysis results with PHQ2 scores of 4 and less

| PHQ-9 | Model 1 | Model 2 | Model 3 |
| --- | --- | --- | --- |
| WYSA | 0.4384  (-2.7218, 3.5987) | 0.2001  (-3.4605, 3.8622) | 0.6383  (-3.5308, 3.8246) |
| Control variables | No | Yes | Yes |
| Number of sessions | No | No | Yes |

N=39
